# Earlier occurrence and increased explanatory power of climate for the first incidence of potato late blight caused by *Phytophthora infestans* in Fennoscandia

**DOI:** 10.1371/journal.pone.0177580

**Published:** 2017-05-30

**Authors:** Veiko Lehsten, Lars Wiik, Asko Hannukkala, Erik Andreasson, Deliang Chen, Tinghai Ou, Erland Liljeroth, Åsa Lankinen, Laura Grenville-Briggs

**Affiliations:** 1 Department of Physical Geography and Ecosystem Science, Lund University, Lund, Sweden; 2 Dynamic Macroecology/ Landscape dynamics, Swiss Federal Institute for Forest, Snow and Landscape Research WSL, Birmensdorf, Switzerland; 3 Department for Research and Development, The Rural Economy and Agricultural Society Scania, Sweden; 4 Management and Production of Renewable Resources, Luke, Natural Resources Institute Finland, Jokioinen, Finland; 5 Department of Plant Protection Biology, Swedish University of Agricultural Sciences (SLU), Alnarp, Sweden; 6 Regional Climate Group, Department of Earth Sciences, University of Gothenburg, Sweden; Agriculture and Agri-Food Canada, CANADA

## Abstract

**Background:**

Late blight (caused by *Phytophthora infestans*) is a devastating potato disease that has been found to occur earlier in the season over the last decades in Fennoscandia. Up until now the reasons for this change have not been investigated. Possible explanations for this change are climate alterations, changes in potato production or changes in pathogen biology, such as increased fitness or changes in gene flow within *P*. *infestans* populations. The first incidence of late blight is of high economic importance since fungicidal applications should be typically applied two weeks before the first signs of late blight and are repeated on average once a week.

**Methods:**

We use field observations of first incidence of late blight in experimental potato fields from five sites in Sweden and Finland covering a total of 30 years and investigate whether the earlier incidence of late blight can be related to the climate.

**Results:**

We linked the field data to meteorological data and found that the previous assumption, used in common late blight models, that the disease only develops at relative humidity levels above 90% had to be rejected. Rather than the typically assumed threshold relationship between late blight disease development and relative humidity we found a linear relationship. Our model furthermore showed two distinct responses of late blight to climate. At the beginning of the observation time (in Sweden until the early 90s and in Finland until the 2000s) the link between climate and first incidence was very weak. However, for the remainder of the time period the link was highly significant, indicating a change in the biological properties of the pathogen which could for example be a change in the dominating reproduction mode or a physiological change in the response of the pathogen to climate.

**Conclusions:**

The study shows that models used in decision support systems need to be checked and re-parametrized regularly to be able to capture changes in pathogen biology. While this study was performed with data from Fennoscandia this new pathogen biology and late blight might spread to (or already be present at) other parts of the world as well. The strong link between climate and first incidence together with the presented model offers a tool to assess late blight incidence in future climates.

## Background

**Fig 1:**
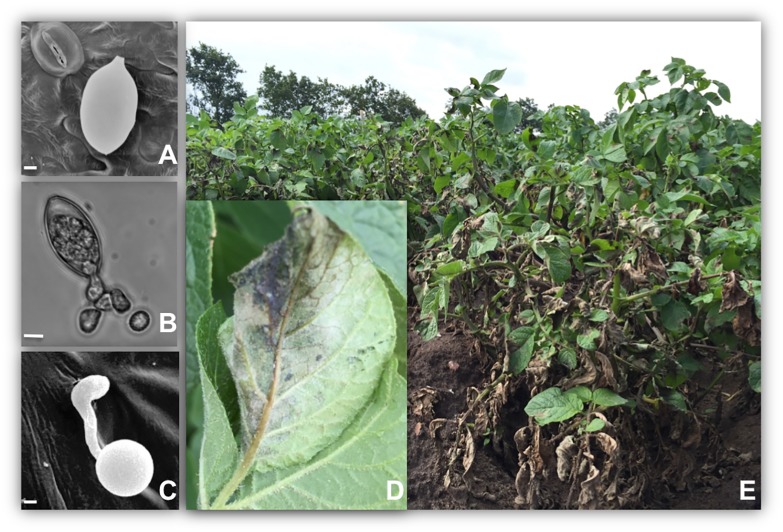
Late blight of potato is caused by the oomycete pathogen *Phytophthora infestans*. A: sporangia are aerially dispersed and deposited on the surface of potato leaves. B: Sporangia release motile zoospores in cool wet conditions. C: penetration of the leaf epidermis occurs via production of appressoria. D (close up) and E: disease symptoms including: water soaked and sporulating necrotic lesions in susceptible cultivar Binjte grown under field conditions, Borgeby, Sweden 2015. Scale bars A and C, 2 μm; B, 10 μm. All photographs: Laura Grenville-Briggs.

Late blight caused by the oomycete *Phytophthora infestans* (Mont.) de Bary, is a devastating disease of potato and tomato (Fig 1). The disease rose to prominence in Europe in the 1840s, where it was responsible for severe epidemics including the Irish potato famine [1,2]. With a centre of origin close to the centre of origin of cultivated Solanum species in Central and Southern America, the pathogen nowadays has a worldwide distribution and is found wherever potatoes are grown [3]. It has been described as a re-emerging disease, since several highly aggressive new isolates have recently been identified around the world. The severity of a disease outbreak is strongly influenced by several factors including the prevailing climate on the pathogen lifecycle, aggressiveness and fungicide resistance within the local pathogen population and the resistance status of the potato cultivar being grown [4].

A predominantly asexual mode of reproduction allows a rapid and dramatic increase in the pathogen population within a susceptible potato cultivar under the right climatic conditions. Aerial asexual spores, (sporangia) (Fig 1A) are able to germinate in free water, either directly via production of a germ tube (at temperatures of 20–25°C) or indirectly via production of motile, wall-less zoospores (Fig 1B), which are produced at lower temperatures (optimally between 10–15°C [5]). Both of these spore types produce specialised penetration structures (appressoria) upon germination (Fig 1C), which allow them to break the plant cuticle and initiate infection [6]. Each sporangium can produce 8–10 motile zoospores, therefore cooler temperatures which encourage indirect germination allow the rapid expansion of inoculum within the field. Macroscopically there are very few symptoms during the first 2 days, making the initial phase of infection difficult to spot in the field. Eventually, water-soaked lesions become visible (Fig 1D and 1E) and *P*. *infestans* can complete the asexual cycle within approximately 4 days under high humidity (all mentioned humidity refers to relative humidity in this study). Up to 300 000 new sporangia may be produced from a single lesion, and these spores can be airborne for up to 40–60 km allowing a rapid and wide-scale spread of the disease in a potato growing region [5,7].

The majority of table potato cultivars grown are susceptible or only moderately resistant, since it has proven historically difficult to combine desirable agronomic traits with durable late blight resistance. Therefore, frequent fungicide sprayings are necessary to protect the crop. The costs of crop losses and damage caused by late blight are estimated at over €1 billion in Europe alone [8].

Fungicides are typically applied weekly throughout the growing season to control the disease; making potato one of the most sprayed crops in Europe. Thus, there is a significant economic and environmental burden to the production of blight free potatoes. The timing of the first application is difficult to determine since the fungicide ideally should be applied before clear symptoms are visible at the site. This has led to the development of a number of different late blight forecasting models, which have been in use for several decades. Many of these models have been built into decision support systems, providing advice about the likelihood and severity of attack, and hence a recommendation as to when to spray the crop. The literature differentiates between late blight forecast and simulation (for a review see [9]), which both require an estimate of the first incidence of late blight. In this study we will concentrate on modelling this first incidence of late blight, neglecting disease severity. The review of Forbes *et al*. [9] concludes that only a few cases showed better disease control with the use of a forecasting system than weekly sprays (and on average fungicide usage was not reduced by use of a forecasting system).

One of the earliest operational forecast models was developed by Wallin [10] based on data collected in ‘blight gardens’. This model sums the hours that plant is exposed to relative humidity above 90% at a certain temperature range starting at emergence. It was later improved and variants for different severities were generated in the SIMCAST model [11]. SIMCAST sums so called late blight units over time starting from plant emergence. It became the basis for a number of applications. While Grünwald *et al*., [12] concluded that SIMCAST was recommending too many fungicide applications for highly resistant varieties, a modification of SIMCAST by [13] resulted in a good performance of the model. Global applications of SIMCAST have been performed by Hijmans *et al*. [14] and Sparks *et al*.[15]. The first study uses current climate data to apply SIMCAST globally to produce a map of the number of required fungicide applications and it also relates the results to predictions by BLITECAST (model based on the estimation of leaf wetness [16]). The second study uses climate projections and evaluates the amount of change in blight units. Both studies use a meta-model to be able to estimate SIMCAST blight units (which depend on hourly data) by using monthly climate data. In our analysis we are only using the blight units which are accumulated by SIMCAST as a predictor for the first occurrence of late blight and not the full functionality which also includes e.g. fungicide weathering. Hence when we write that we compare our results with SIMCAST, we actually compare our results to the blight units accumulated by SIMCAST.

A number of field trials in different countries have over the last decades monitored the occurrence of late blight and the effectivity of the applied fungicide. A summary of 30 years (1983–2012) of observations of late blight occurrence in southern Sweden showed that on average the first attack occurred one day earlier each year during the investigation period [17]. Hence compared to the conditions in the early 80’s fungicide applications need to start a month earlier nowadays resulting in 4–5 more applications required to control the disease. This study also showed that the harvest in untreated populations decreased due to a higher disease severity over time. Hannukkala *et al*.[18] also reported earlier late blight outbreaks in the period from 1998–2002 compared to the preceding decades in Finland. Outbreaks started on average 2–4 weeks earlier and were correlated with a climate that was more conducive to late blight, but also to a lack of crop rotation and the occurrence of sexually reproducing pathogen populations [18].

Here we use data from sites in Sweden and Finland and assess how well the sums of blight units used in the most common current model SIMCAST predict the timing of disease outbreaks. Since fungicide applications are usually recommended to commence 2 weeks before the first incidence of late blight, precise predictions of the onset of disease are crucial to minimise fungicide applications, the risk of loss of harvest and the risk of pathogen resistance to fungicides (which can be driven by overuse of such products). Using SIMCAST as a starting point for disease prediction modelling, we investigate how the first incidence of late blight can be related to, and hence predicted by, the weather in an interdisciplinary approach.

## Materials and methods

### Field trials

The monitoring of late blight outbreaks and epidemic development was carried out at two sites in Finland, Lammi (61°05’ N; 25°01’ E) and Jokioinen (60°49’ N; 23°30’ E), from 1988 to 2011, and at three sites in Sweden, Mosslunda (55°58’ N, 14°6.3’ E) Borgeby (55°45’ N, 13°2.3’ E) and Lilla Böslid (56°36’ N, 12°57’ E), from 1983 to 2012.

The data were collected from untreated control plots from fungicide efficacy trials (cv. Bintje) and Bintje plots in variety trials with no chemical late blight control. All trials had completely randomized block design comprising four replicates each. The trials were planted between the second week of May and the first week of June depending on the weather conditions in each year and site. All cropping measures were conducted in the same manner as in conventional potato cultivation. Commercial, certified seed potato (in Sweden using local certification schemes [19] based on the EPPO standard and in Finland using the EPPO standard [20]) was used in the trials, hence the seed potatoes are certified to be free of late blight, viruses and other pathogens. Crop rotations of at least 5 years between repeat plantings of seed potato were performed at all sites and strict late blight chemical control schemes were adhered to. Normally there is no late blight in seed potato production fields, due to long intervals between repeat plantings of potato and strict fungicide applications, thus oospores are not usually detected in these fields.

The planting density was 4 seed tubers/m and row space was between 75 and 80 cm. The potato was fertilized at planting with compound fertilizer (N 70–80 kg/ha) and later supplemented. Weed control was done with standard herbicides before potato emergence. The trials at Lammi and the trials in Southern Sweden were conducted on sites where no potato was grown in three preceding years while variety trials at Jokioinen were conducted in fields with very intensive potato cropping history.

The trial plots were monitored for late blight occurrence at intervals of 3 to 6 days each year. The date of the first blight occurrence and total disease incidence was recorded for the day when first late blight symptoms were detected in each observation plot. Natural infection of potato late blight occurred in all field trials without the need for artificial inoculation.

A key covering very small infection levels was used, but otherwise assessment was consistent with commonly used scales [21]. An attack of 0.01% corresponded to one blight-spot per 50 plants, 0.1% one blight-spot per plant, 1% up to 10 blight-spots per plant, 5% about 50 blight-spots per plant and 10% about 100 blight-spots per plant. Other late blight symptoms on leaves, such as infected new shoots, stem blight and leaf stem blight, were given values to estimate the corresponding damage. To estimate number of days after planting (DAP) at the onset of the epidemic or when the first attack occurred, a reduction of DAP by three days was made if the attack at the first assessment was < 0.01%, by five days if the attack was > 0.01–0.099%, by seven days if the attack was 0.1–1.0% and by nine days if the attack was > 1%. This method is based on the average development rate of the disease and is a modification of the EPPO scale [22] used extensively for European late blight monitoring (see [23] and Fig 2 in [17]) and allows comparison with previous studies (e.g. [17]).

### Meteorological data

Hourly temperature and humidity data was required in this work since SIMCAST sums hourly late blight units. However, only a few sites in the study area had instrument observation and none of these covered the whole analysis period. Therefore, other options were required. One such option is to utilize reanalysis datasets. Unfortunately, commonly available reanalysis datasets have a horizontal resolution of around 250km and the temporal resolution is 6 hourly. A downscaling in time and space was thus needed to fill the gap. For this purpose, a regional meteorological model, i.e. the latest version of the Air Pollution Model, TAPM version 4, [24], was utilized as a tool for the required dynamic downscaling. TAPM is a three dimensional nestable, prognostic meteorological and air pollution model. This model has been previously applied in Sweden to successfully reproduce hourly temperature and wind in Gothenburg area [25]. In this study, 6 hourly surface pressure, geopotential height at various pressure levels, zonal and meridional winds, air temperature and specific humidity from NCEP/NCAR reanalysis during the period 1980–2014 [26] were utilized to drive TAPM. The domain of TAPM is centred at the location (60^o^N, 19^o^30’E) with 45 x 45 horizontal grids at 30km spacing. The lowest five of the 30 model vertical levels are 10, 25, 50, 75, 100m a.g.l. (above ground level), with the highest model level at 8000m a.g.l. Simulated hourly near surface air temperature and relative humidity have been evaluated using hourly instrument observation at the three Swedish sites, Borgeby (2007.05.12–2014.12.31), Mosslunda (2011.05.11–2014.12.31) and Lilla Boslid (2009.01.01–2014.12.31). Results show that TAPM has a good model performance in reproducing hourly variation of related climate variables. More than 91% of the observed variation of hourly near surface air temperature is explained by the TAPM simulations. TAPM simulations can also explain about 45% of the observed variation of hourly relative humidity. This shows that the simulated meteorological fields are reasonable. This hourly data was subsequently also used to generate daily data if required for later analyses.

### Statistical analyses: SIMCAST

**Fig 2:**
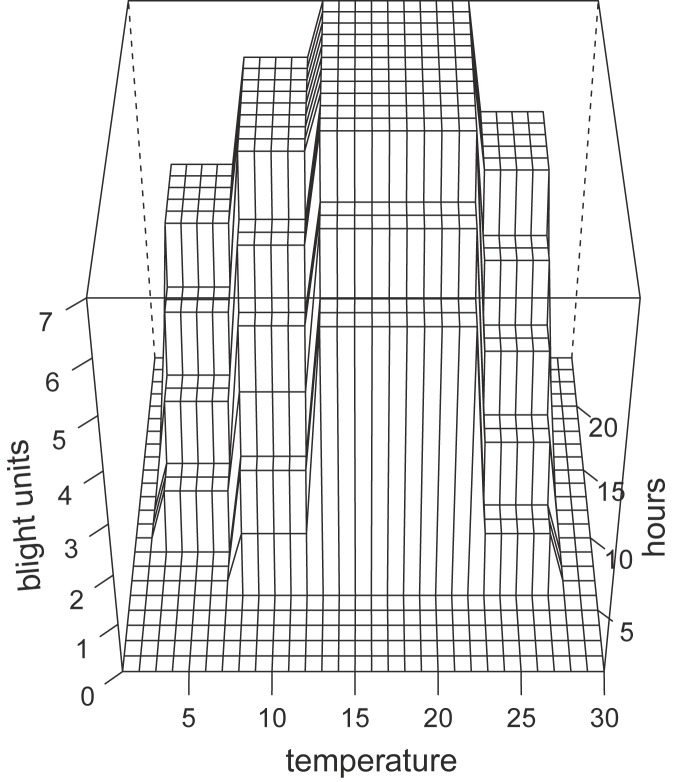
Graphical representation of the SIMCAST lookup table. The number of blight units to be added for each moist period (relative humidity ≥ 90%) depends on the mean temperature (in degree Celsius) during the period and the length of the period in hours.

SIMCAST uses cumulative blight units value over time from host plant emergence. The units depend on consecutive hours of high relative humidity (≥90%) and the average temperature during these periods. Different late blight units are assigned depending on the cultivar resistance of which the model differentiates susceptible, moderately susceptible and moderately resistant cultivars. Since the SIMCAST model [11] was developed to be easily applicable before the current wide availability of computers, it was formulated in the form of a lookup table and was intended for prediction of first occurrence of late blight to time the application of fungicides. The lookup table listed the amounts of late blight units to add for each period of high humidity (≥ 90%). In Fig 2 we visualised the table in a graph for the susceptible cultivar like Bintje, the cultivar used in this analysis.

To be able to use the SIMCAST model in our analysis we needed a continuous function to represent the lookup table of SIMCAST. Fundamentally the SIMCAST model is simulating late blight disease development. Therefore, we used a common approach to simulate the temperature dependence of the growth of plants, the beta function [27] which uses the three temperatures characteristic for the simulated species, the minimum temperature *T*_*min*_, the maximum temperature *T*_*max*_ and the optimum temperature *T*_*opt*_ for growth (in our case disease development; Eqs 1 to 3).

$$\alpha =\frac{\ln\left(2\right)}{\ln(\frac{T_{\max}-T_{\min}}{T_{\mathrm{opt}}-T_{\min}})}\;|T_{\min}<T_{opt}{<T}_{max}$$ (1)

$$\beta =0\;|T<T_{min}\;or\;T>T_{max}$$ (2)

$$\beta =\frac{{2(T-T_{min})}^{\alpha }{\left(T_{opt}-T_{min}\right)}^{\alpha }-{\left(T-T_{min}\right)}^{2\alpha }}{{\left(T_{opt}-T_{min}\right)}^{2\alpha }}\;|\;T<T_{max\;}and\;T{>T}_{min}$$ (3)

This temperature dependence function is also used as a time dependent covariate in the further analysis (co-variate number 10; Table 1).

**Table 1:** Covariates used in the statistical analysis. All variables except Location are time dependent.

Co-variate number	Comment
1	Year: used to test whether a temporal trend which can not be explained by the climate can be detected
2	Country: a variable coding whether the experimental sites were in Sweden or Finland
3	Station: a variable coding the location of the experimental sites (the data was collected in 5 locations)
4,5,6	Blight units calculated by SIMCAST based on a threshold of 0%, 50% or 90% rel. humidity. The originally suggested threshold is 90% rel. humidity.
7	The cumulative sum of the daily mean temperature
8 9	Minimum winter temperature between October and February of the previous winter The cumulative sum of the daily mean relative humidity
10	The cumulative sum of the temperature dependent growth (beta temperature)
11	The cumulative sum of the temperature dependent growth multiplied with the relative humidity

The increase of blight units with length of the period is sigmoidal, hence we approached this with a sigmoidal function (Eq 4).

$$s=\frac{a}{1+e^{b+hc}}$$ (4)

Here *s* denotes the sigmoidal time response, h stands for the number of hours that a single period lasts and *a*, *b* and *c* are parameter to be fitted from the SIMCAST lookup table. The optimal values for *a*, *b* and *c* as well as the temperature related parameters are: *T*_*min*_ = -1.9453, *T*_*opt*_ = 17.1268, *T*_*max*_ = 29.6298 *a* = 7.3250, *b* = 4.6006, *c* = -0.5270 (all temperatures in degree Celsius, a,b,c are unit less).

**Fig 3:**
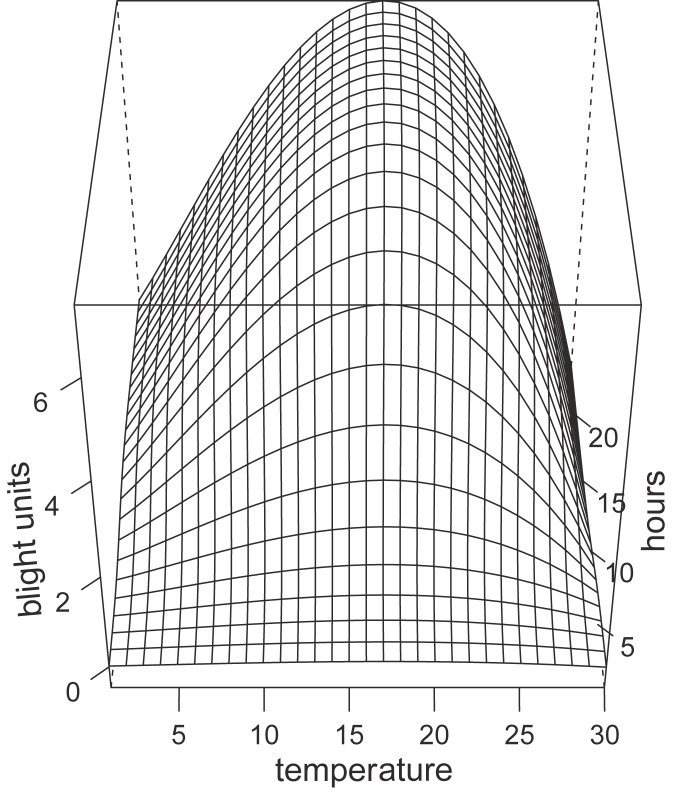
Continuous representation of SIMCAST based on a beta function along the temperature gradient and a sigmoidal function along the hour gradient. The number of blight units to be added for each wet period (relative humidity≥ 90%) depends on the mean temperature (in degree Celsius) during the period and the length of the period in hours.

We combined the beta function (along the temperature axis) with the sigmoidal function (along the hour axis) multiplicatively and fitted a combined model to the values given by SIMCAST (see Fig 3).

SIMCAST is typically summing up blight units for periods of high humidity (≥ 90%). Based on preliminary results we hypothesized that progression of late blight disease symptoms could occur even during periods of lower relative humidity. To test the performance of lower thresholds we also calculated blight units based on thresholds of 50% and 0% relative humidity.

### Statistical analyses of late blight occurrence

The first occurrence of the late blight has been modelled using a Cox proportional hazard model for survival [28] with time dependent and time independent covariates. This model type is the most commonly used multivariate approach for analysing survival time data in medical research [29]. It is basically a regression model describing the relationship between the event incidences (in our case late blight detection) expressed as a hazard function and a set of co-variates (in our case for example blight units). The general form of the model is given in Eq 5.

$$\lambda (t)=\lambda_{0\;}(t)\cdot e^{\left(b_{1}x_{1}+b_{2}x_{2}+\ldots +b_{n}x_{n}\right)}$$ (5)

Here the hazard function λ(t) depends on the covariates x_1_, x_2_,… x_n_, and their impact is measured by the estimated coefficients b_1_, b_2_,… b_n_. This general form assumes a time independence of the covariates (for example in our case the location is a time independent covariate). The model is expanded for our analysis to time dependent covariates by replacing the time invariant covariates x with a time dependent form x(t).

Since this model is semi-parametric it does not require the choice of a certain probability distribution in advance.

For all sites, daily values of the time dependent covariates listed in Table 1 were used in the analysis. Since we assumed the development of the pathogen to start in the presence of potato leaves, we started the time series containing the covariates at the emergence of the potato. Due to the unavailability of actual emergence data for a large proportion of the data we estimated the emergence of the potato to be at the day where the growing degree day sum above 2 degree Celsius are above 220. Using a growing degree sum to estimate potato emergence was suggested by Pulatov *et al*. [30], and the value of 220 has been estimated using the years for which emergence data was available. While only climate data starting from the day of emergence is used in the analysis, the age of the potato until first occurrence of late blight is estimated in days since planting. Though for the further analysis daily values were used, the calculation of the daily late blight units (co-variate 4, 5 and 6 in Table 1), was performed with hourly values.

In an initial screening we used more variables than the variables listed above, including using hourly variables instead of daily variables. However we excluded them in a second stage due to high correlation to at least one of the variables in Table 1. Initial tests with climate data supplied as daily mean values instead of cumulative sums have also shown that these values lead to no improvement of the predictive power for the occurrence of late blight. A file containing all meteorological data covering the time from the emergence to the first occurrence of late blight has been collated and is available from the DataGURU server (dataguru.lu.se) with the DOI 10.18161/late_blight.201703. The description of the data is given in S3 Fig.

### Temporal changes in climate response of late blight

To test whether the climate dependence of the first day of occurrence of late blight has changed with time we estimated the proportional hazard model using a moving time window of 10 years.

For each of the 10-year periods we estimated the model performance. Before applying the time window we separated the data by country of origin, since the sites within a country are considered to be close enough to not exhibit different temporal patterns. For this test we used all variables listed in Table 1 since we were interested in the maximum explained variability.

To show how the model can be used to operationally predict the first incidence of late blight, we took weather data from a single year and displayed the resulting hazard rates over time. Finally we evaluate the robustness of the estimated parameter by using a bootstrap method where we discard 40% of the data and analyse the variability of the estimated parameter.

### Ethics statement

The trials in Sweden were carried out on experimental stations owned by the conductor of the experiment (The Rural Economy and Agricultural Societies) or at rented farms. The farmers, (the private land-owners) gave permission to conduct the studies on these sites. The Finnish experiments were carried out at sites owned by the Ministry of Agriculture in Finland and dedicated for experimental purposes. The rights of individual farmers or protected species were not violated. The field studies did not involve endangered or protected species.

## Results

### Occurrence of late blight over the whole investigation time

**Fig 4:**
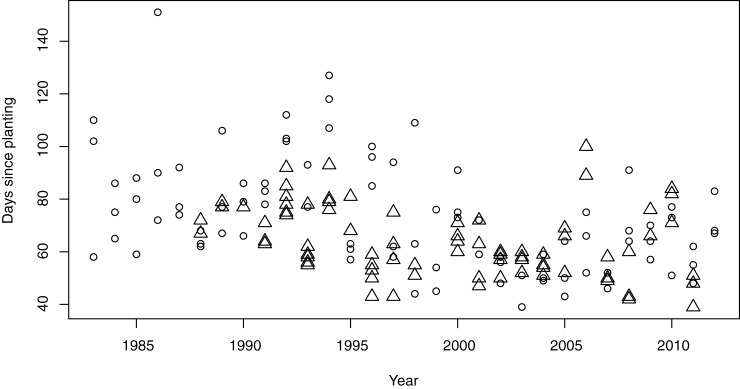
Occurrence of late blight over the investigation time in Finland (triangles) and Sweden (circles).

The number of days after planting at which late blight occurred during the different years in our study is illustrated in Fig 4.

To test whether there has been a general trend of earlier occurrence of late blight over the whole investigation time, we generated a Cox proportional hazard model [31] using only year as a predictive variable. It resulted in a p-value of 2.7·10^−6^ with an exponential coefficient of larger than unity (1.047) indicating a significant earlier occurrence of late blight in later years.

**Fig 5:**
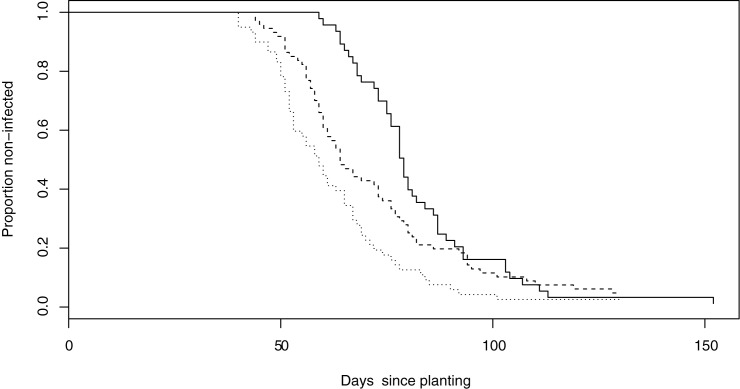
Proportion of non-infected potato trials separated in three 10 year periods.

In Fig 5 we plotted the proportion of non-infected trials versus the days since planting for three 10-year periods of our data. It shows that the days since planting until a 50% chance of infection is reached have decreased by little less than a month from the first decade to the last.

### Temporal changes in climate response of late blight

**Fig 6:**
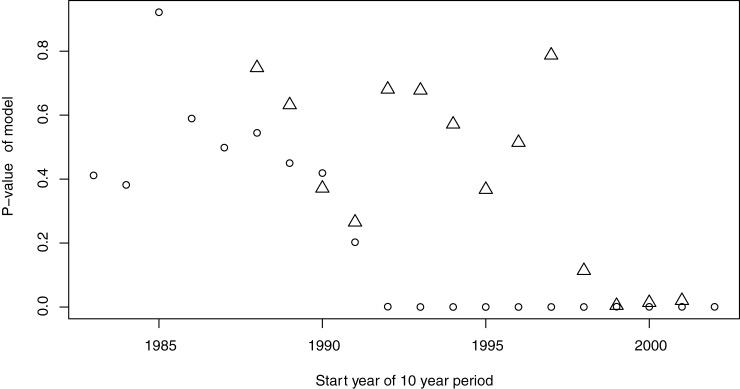
Predictability of occurrence of late blight in Finland (triangles) and Sweden (circles) based on a moving window of 10 years expressed in the p-value of the model containing all parameters listed in Table 1. Please note that the data series for Finland starts in 1990 and ends already in 2011.

We used a 10-year moving window of data and estimated the predictive ability of the Cox proportional hazard model using the p-value of the resulting model. As displayed in Fig 6, the predictive ability based on 10 years of data is very low before 1992 in Sweden and also very limited before 1999 in Finland. After these years, climate becomes a good predictor of the first occurrence of late blight. Given these results we hypothesise a change in the response of the pathogen to the climate and will in the following analysis only use the data that corresponds to the time where climate is a good predictor. Since the moving window has a size of 10 years we discard the Swedish data from before 1994 and the Finnish data from before 2001. As the model reports the relationship averaged over the whole time period, the strong relationship in the last years might be outweighing a weak relationship in the start of the period.

### Response of late blight to climate

To find the most appropriate model for late blight occurrence, we started by evaluating the predictive power of the variables listed in Table 1. In the following we evaluated the predictive power and estimate coefficients for three cases: Swedish data only, Finnish data only and a pooled dataset. This allowed us to identify to what extent the relationship could be generalised. The listed exponential of the estimated coefficient (exp(coeff)) indicate the direction of the relationship, where a value larger than unity indicate a positive relation to the hazard rate, and a value below unity a negative relationship. For example the exponential coefficients for the variable “Year” are above unity showing that in later years the hazard rate was higher and hence late blight occurred earlier than in earlier years. We selected the climate composites based on previous knowledge of risk factors for late blight occurrence. For all tested variables a positive relationship was found (in case of a statistically significant relationship), hence an increase in any of the variables would lead to an increase of the hazard rate and hence we would expect an earlier outbreak of late blight.

All models in Table 2 contain only one parameter (even it is a combination of several co-variates it is treated as one parameter since only one coefficient is estimated).

**Table 2:** Predictive power and coefficient of the covariates without combinations. Acronyms: temp.: temperature; rel.hum.: relative humidity; min.: minimum. An exp(coeff) value larger unity indicates a positive relationship.

Co-variate [number, description]	p-value Sweden	exp (coef)	p-value Finland	exp (coef)	p-value pooled	exp (coef)
1. Year	5.14·10^−3^	1.069	n.s.		2.80·10^−2^	1.043
2. Country	n.a.		n.a.		n.s.	
3. Station	n.s.		n.s.		n.s.	
4. SIMCAST with threshold rel. hum. >90% (standard)	n.s.		n.s.		n.s.	
5. SIMCAST with threshold rel. hum. >50%	8.51·10^−6^	1.028	5.33·10^−3^	1.020	7.72·10^−8^	1.025
6. SIMCAST with threshold rel. hum. >0	2.59·10^−8^	1.051	1.73·10^−2^	1.018	4.76·10^−7^	1.029
7. Cumulative sum of mean daily temp.	5.80·10^−5^	1.008	n.s.		2.35·10^−4^	1.005
8. Minimum winter temp (Oct-Feb)	n.s.		0.044	1.199	n.s.	
9. Cumulative sum of mean daily rel. hum.	3.91·10^−4^	1.002	1.34·10^−3^	1.002	1.31·10^−6^	1.002
10. Cumulative sum of mean daily beta temp.	5.95·10^−9^	1.434	1.81·10^−2^	1.131	2.58·10^−7^	1.233
11. Cumulative sum of mean daily beta temp. multiplied with rel. hum.	7.72·10^−6^	1.003	7.21·10^−4^	1.002	3.05·10^−8^	1.002

Additionally we estimated p-values for the covariates combining model 1 and model 11 and model 8 and model 11, see below.

Table 2 reports uncorrected p-values. We are aware that the p-values in the table constitute a case of multiple testing, since several p-values are generated from a single dataset. In total we generated 36 p-values, so according to the most conservative correction for multiple testing, i.e. the Bonferroni correction, the significance level would have to be changed from 5 ·10^−2^ to 5 ·10^−2^ / 36 = 1.3 ·10^−3^, hence except for the variable “Year“, and some variables tested for the Finnish data only, all reported p-values remain significant even if we perform the most conservative correction for multiple testing.

As shown in Table 1, the cumulative blight units used by SIMCAST have a high predictive value if not the original threshold of 90% relative humidity is applied but a lower one of 50% or 0%. Since the vast majority of the measured values of relative humidity are between 50% and 100%, (S2 Fig) the performance of the models based on either 50% or 0% relative humidity are very similar.

The model based on the cumulative sum of temperature multiplied with relative humidity resulted in an estimated exponential coefficient of 1 + 2.182 ·10^−3^ with a standard deviation of 3.94 ·10^−3^ and a concordance of 72% indicating that in 72% of pairs of cases the case with the higher risk had an event before the case with the lower risk.

Hence the final model of the hazard rate based on Table 1 for the combined data set is:

$$\lambda (s(t))=\lambda_{0\;}(t)\cdot e^{\left(0.0021820\cdot \;s(t)\right)}$$ (6)

$$s(t)=\sum_{i=1}^{t}rh(t)\cdot \beta (T(t))$$ (7)

With *λ(s(t))* indicating the probability of the incidence at day *t* being dependent on the cumulative sum *s* of the product of the relative humidity at day *t* and the *β*-function of the temperature *T* at day *t*.

Similarly to the variable “Year” in the dataset covering the full time span, the “Year” variable also showed a significant positive relationship to the occurrence of late blight (at least if no correction was applied).

When the response of late blight occurrence was tested using a model containing the cumulative sum of temperature multiplied by relative humidity with year as a variable parameter, (model 1 and model 11) year was not found to be a significant predictor of late blight occurrence, showing that the earlier onset of late blight (over the late period of time tested) can be explained by climate alone while there is no additional effect of the year.

The two additive models containing the cumulative sum of temperature multiplied with relative humidity and the variable winter temperature (model 11 and model 8) also showed that in these models only the first parameter resulted in a significant p-value, even for the Finnish data, which showed a significant response to the parameter winter temperature when tested on its own. Hence the minimum winter temperature might be correlated to some extend to the cumulative sum of temperatures, resulting in some explanatory value, but in combination with the cumulative sum of temperatures, no improvement of the model is reached (this has also been indicated by the AIC values of model 11 and a model where winter temperature is added which are identical at 7 digits of precision).

**Fig 7:**
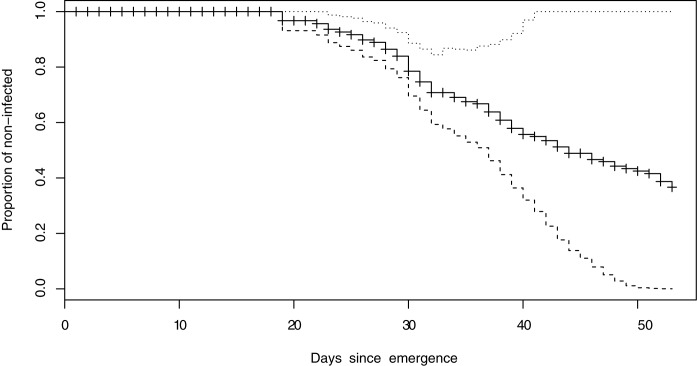
Example prediction of incidence of late blight based on weather data in the year 2010 at a single site. The central cross-line indicates the estimated hazard rate while the upper and the lower confidence intervals are given as dotted or broken lines respectively.

To demonstrate how our model can be used we applied it to weather data from a single year (since this is not an evaluation we used a year which has already been used in the parameterisation) in Fig 7.

### Model robustness

**Fig 8:**
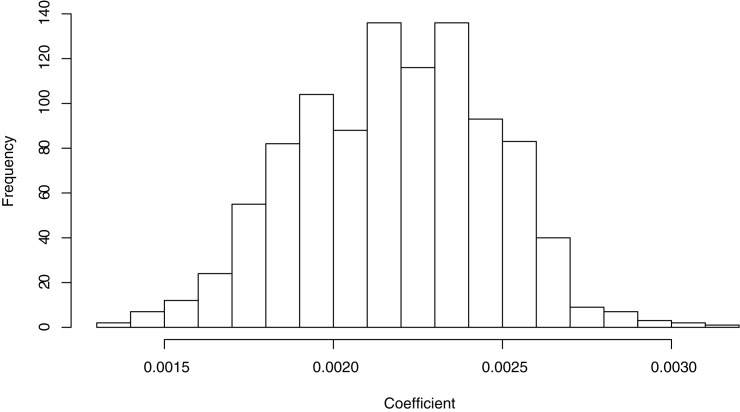
Evaluation of parameter robustness. The coefficient is estimated from a proportion of the data sets excluding ca 40% of the data using a bootstrap method.

The model is fitted with only one parameter. Here we assess how robust this parameter assessment is. We used a bootstrap method where we 1000 times randomly exclude two sites and 3 years, which on average ends up as ca 40% of the data. We estimated the single parameter and display the spread of the estimated parameter in Fig 8.

The target variable (day of late blight incidence) is estimated using the method described before, based on scorings which were done weekly or even more frequently. Hence it carries a certain uncertainty (as all other variables as well). To test the robustness of our result, i.e. that the model using the cumulative sum of the product of the relative humidity and the beta temperature is the best suited model, we randomised the day of first occurrence in the dataset by adding a random (uniform) variability of 5 days. This is a rather high estimate of the uncertainty. We performed 1000 randomisations and estimated all p-values (only for the complete dataset) for each randomisation. In none of the 1000 randomised datasets was the best model different from the one that is found in the original dataset. Hence we consider the result valid given the uncertainty in the estimated first day of occurrence (see S1 Fig for distributions of p-values).

## Discussion

Our study has two main findings. Firstly, we find that the predictability of the incidence of late blight has changed over time. In the Swedish sites the occurrence before 1994 and in the Finnish sites before 2001 is rather erratic, whilst after these years the first incidence has a strong link to weather. The second finding is that the inactivity of late blight at relative humidity below 90%, which is assumed in a number of predictive models, seems to be wrong. We find a rather linear increase of activity (in our case hazard rate) with increasing humidity.

### Changes in the predictability of late blight over time

As reported by both Wiik [17] and Hannukkala *et al*. [18], our analysis of both the Swedish and Finnish data showed a significantly earlier incidence of late blight during the later years of trials compared to the earlier period. Surprisingly, prior to 1994 for the Swedish data and 2001 for the Finnish data, the weather is not a good predictor of the likelihood of an outbreak of potato late blight (using the weather derived variables of this study). However after these dates there is a strong link between weather and late blight incidence. This suggests a change in the growth-response of the pathogen to the climate or a change in pathogen biology to allow earlier infections, more closely linked to the weather. We believe that a change in the host plant response to allow earlier infections is unlikely, since all of our data is based on the use of a single highly susceptible potato cultivar, Bintje, which has been used for the field-testing of fungicide efficacy during the entire time period we analysed and has no resistance to the pathogen. However, particularly in Sweden there has been a tendency towards a consolidation of the potato farming industry, so that fewer larger farms dominate production now compared to the situation 30 years ago (Eriksson et al 2016). This may mean that inoculum pressure is higher in regions close to larger potato farms than before, however our data were collected in regions with already intense potato production and whilst ownership of those farms have changed during the period we have no data on specific changes in inoculum levels in relation to these changes in potato production. Similarly although potato cultivars favoured by industry may have changed during this time period, we collected data from a single highly susceptible potato cultivar grown in rather small experimental plots throughout our study, and thus we believe changes in cultivar usage over time have had little or no impact on our data.

Although the initial migration of *P*. *infestans* from Mexico and the Andes into Europe occurred during the Irish potato famine in the 1840s, it is now evident that a second major migration event occurred in the winter of 1976/7 when a large shipment of contaminated table potatoes was imported directly into Europe from Mexico [32], introducing new strains once again into Europe. The first introduction of *P*. *infestans* into Europe brought only a single mating type (A1). As *P*. *infestans* is a heterothallic oomycete, it requires two different mating types for sexual reproduction. Since the second mating type (A2) was only introduced into Europe during the second migration of this pathogen, all incidences of the disease in Europe before 1980 were caused by asexually reproducing pathogen populations. Sexual reproduction in the oomycetes leads to production of an oospore, which serves as both a source of variation via sexual recombination and as a survival structure, protected by several thick layers of cell wall and a large reserve of lipids and other energy molecules [5]. Oospores, unlike their asexual counterparts can remain in the soil after potato harvest and survive extreme conditions for extended periods and can therefore be an early infection source [33].

The first reported occurrence of an A2 *P*. *infestans* isolate in Sweden was in 1985 [34]. A2 isolates were found in Finland approximately seven years afterwards in 1992 [35]. Interestingly, the change in first incidence detected by our model comes 9 years after the first report of the presence of the A2 mating type in Sweden and 8 years after the same report in Finland. This delay in changing incidence may represent the time needed for sexual reproduction to become established in these countries and for the population dynamics to change, but further experimental analysis will be needed to test this hypothesis.

By the late 1990s Nordic populations of *P*. *infestans* were showing the hallmarks of sexually derived genetic variation [36,37]. Andersson *et al*., (1998) identified the presence of both mating types within the same fields in Sweden for the first time in 1996 and by the early 2000s oospore production was identified in approximately one third of tested fields in Southern Sweden [38] and Finland [39]. Thus, the available evidence from the Nordic region strongly supports the identification of both mating types of *P*. *infestans* in Sweden and Finland and furthermore, regular sexual reproduction among *P*. *infestans* populations in this region appears to be on-going [40]. The occurrence of sexually derived isolates strongly correlates with a stronger link to the weather and earlier disease outbreaks during the time period we investigated, which might be explained with the larger availability of oospores which can easily overwinter in the soil in this time period.

Given that we have no data concerning whether historical, or current, infections were caused by oospores or not, we cannot conclude that the change that we see in the response to the climate is due to sexual reproduction, within the pathogen resulting in oospore derived infections. It is possible that the observed changes are caused by different variants of *P*. *infestans* dominating the pathogen population at the experimental sites. Given that sexual reproduction (which allows fast recombination of genes) has been observed to occur in Fennoscandia, and that *P*. *infestans* has a vast potential for fast adaptation to its external environment [41] variants more adapted to the current climate (of rather mild winters and early springs in the last 2 decades) might have evolved and be currently dominating Fennoscandinavian late blight populations. However, this hypothesis remains to be tested.

### Relationship between disease occurrence and relative humidity

Although the effects of relative humidity on late blight are not well studied, Minogue and Fry [42] concluded that there was no significant effect of altered humidity (in a range from 40–88%) on sporangial viability. However, many late blight prediction models, such as SIMCAST and its derivatives, presume a threshold of 90% relative humidity for late blight infection to occur and spread. Such models are based on historical data such as the study by Harrison and Lowe [43] which demonstrated that *P*. *infestans* sporangia were formed abundantly at 90–100% relative humidity with a wind speed of 0.3 x 10^−3^ but were not formed at all when the humidity dropped to 80 or 85%. However, since it has recently been shown that local populations of *P*. *infestans* exhibit a high level of adaptation to temperature, [44] it is highly likely that modern *P*. *infestans* isolates have adapted to a wider relative humidity range than the single clonal isolates used by [42] and [43]. In fact, given the highly heterogeneous *P*. *infestans* population structure present in the Nordics [40] and the fact that late blight infections derived from mixed infections influence the genotypes of the competing strains both positively and negatively [45] it is highly likely that some strains have adapted to higher or lower humidity conditions. This is also likely to have influenced the timing of the infections, and competition for host plants between genotypically diverse strains of *P*. *infestans* in the field, may be an important factor in driving earlier field infections. Therefore, we can conclude that if variable relative humidity requirements for successful infection are taken into account (including our observed linear relationship between humidity and first incidence of late blight) existing prediction models might significantly improve their prediction capabilities.

The infection mechanism of *P*. *infestans* requires free water on the leaf or very high humidity to allow production and spread of sporangia and zoospores [5]. Studies of the effect of climate change on related foliar pathogens, the downy mildews, have therefore investigated not only the effect of changes in mean temperature but also how dew formation at certain parts of the day changes and hence affects future growth conditions for downy mildew [46,47].

The fact that we have not found a stronger link between the phenomenon of late blight occurrence and the weather variables that we investigated at an hourly scale versus a daily scale might result at least partially from the high variability in the simulated relative humidity data compared to the measured data. It can however also be seen as an indication that the daily relative humidity is a proxy for the number of hours at which free water persists on the surface of leaves.

Our simulated relative humidity has a larger error than the simulated temperature. This is to be expected because it is much more difficult to accurately measure relatively humidity than temperature (see e.g. [48]). Furthermore, in the model, relative humidity is calculated based on temperature (for saturated humidity) and absolute humidity, which depends on surface evaporation and atmospheric transfer of moisture. Thus, the accuracy of the simulated relative humidity not only depends on evaporation and moisture transfer which are difficult processes to be modelled, but also relies on the accuracy of the simulated temperature. Since errors in temperature prediction also propagate into the calculated relative humidity, the simulated relative humidity is expected to be less accurate than the simulated temperature. Given the resolution of the model (30 km) and the local measurements at the two sites, as well as the fine temporal resolution that we have used (hourly), the comparison can still be considered reasonably good.

To validate the usefulness of the simulated weather data for our study, we checked that the distribution of simulated temperature and relative humidity behaved as expected (see S1 to S3 Figs). Given that both sensitivity analyses (the one that added variability to the first date of occurrence and the one that looked at the bootstrapping method, estimating the variability in the estimated parameter) showed that the our result is very robust, we have no reason to believe that the uncertainty in the estimated relative humidity is influencing the validity of our main finding that the response of the pathogen to the weather has changed. It will however certainly add to the uncertainty in the estimated parameter values of the final model. Currently we have no other way of investigating this uncertainty but once longer times series of measured data are available this uncertainty range should be quantified. As we are not estimating the precise day of incidence of late blight but a hazard rate (which is not binary but increases over time), we can not directly compare the occurrence of late blight with our model results. By performing this bootstrapping we evaluate how generally the relationship between the co-variates and the hazard rate is expressed across our dataset.

One other factor that should conceptually influence the occurrence of late blight is the minimum winter temperature, since a strong frost should lead to a limited survival of late blight. However we could not detect an effect of winter temperature on late blight incidence using our models. We therefore have to conclude that, in our dataset, minimum winter temperature is not a significant determinant of late blight incidence, which might be caused by the relatively mild winters of the last two decades.

However, even if winter temperature does effect infection incidence it might be hard to detect in the data given that within the experiment (similar to common agricultural practices) a certain amount of crop rotation is practiced, hence oospore infections might actually result from an infected plant two or more years ago in which case the relationship to the winter temperature of the last year might not be detectable anymore. Along with oospore infections we expect a large number of the infectious propagules to arise from asexual propagation and thus air and water borne spores. Given the mixture of inoculum sources and the lack of precise information on the crop rotation of the historical experimental sites, we were not able to specifically analyse these effects further.

### Model validity and applicability

The best suited model only estimates a single parameter, hence we refrain both from an optimisation of the number of parameter (e.g. using the Akaike criteria [49]) as well as an analysis of correlation between parameters (in our case relative humidity and temperature) since such an correlation would only be problematic if we would introduce parameters for each new variable.

As we are not estimating the precise day of incidence of late blight but a hazard rate (which is not binary but increases over time), we can not directly compare the occurrence of late blight with our model results. By performing this bootstrapping we evaluate how generally the relationship between the co-variates and the hazard rate is expressed across our dataset.

By plotting the distribution of the estimated parameter, using a bootstrap procedure we show that the data is homogeneous and has a unimodal Gaussian shape. Otherwise the distribution would have several local maxima corresponding to parameter values estimated from different subsets of the data.

Our model is currently based on five sites in two Nordic countries, and we have no indication that our model would not be applicable in other areas, which have similar late blight population dynamics. Since the experiments were conducted with one highly susceptible variety of potato (Bintje), we expect our model to be rather overestimating the risk of late blight compared to other varieties, however this is in our case a good property of the model given that the loss of the harvest is at risk. Apart from the variety of potato used by the farmer, there are other factors that should be taken into account when advising the application of fungicides. Decision support systems should not only estimate the risk of first infection but also the severity, which we have ignored in this study to avoid a too high complexity of the subject, as well as economic factors such as the cost of the spray.

At a low risk of the incidence the profitability of applying the fungicide changes with the price of the fungicide, the cost of the application as well as the market value of potato. All of this needs to be taken into account when designing a decision support system, thus having a reliable estimate of the first occurrence is only the first step.

A comparison of the number of fungicide applications at a country scale by [15] showed a non-significant correlation of SIMCAST results with the observed amount of fungicide application, however given that the different countries might either over or underutilise the use of fungicides based on discrepancies in knowledge or limited resources to acquire fungicide this is not unexpected.

## Conclusions

Given that the incidence of late blight occurs now on average 30 days earlier compared to the 80s and that severity of late blight has increased, more research should focus on increasing the accuracy of decision support systems and more resistant varieties. This will ensure that the global production of potato will not suffer in the next decades. Our finding is in line with the review by [50] that points out that at scales relevant to climate change, accelerated evolution and changing geographic distributions will be the main drivers of change for pathogens and thus are important considerations for future disease control strategies.

## Supporting information

S1 Fig (PDF)

S2 Fig (PDF)

S3 Fig (PDF)

## References

[1] Turner RS. After the famine: Plant pathology, Phytophthora infestans, and the late blight of potatoes, 1845–1960. Hist Stud Physiol Biol Sci. 2005;35: 341–370.

[2] Zadoks JC. The potato murrain on the European Continent and the Revolutions of 1848. Potato Res. 2008;51: 5–45.

[3] Goss EM, Tabima JF, Cooke DEL, Restrepo S, Fry WE, Forbes GA, et al The Irish potato famine pathogen Phytophthora infestans originated in central Mexico rather than the Andes. Proc Natl Acad Sci U S A. 2014;111: 8791–8796. doi: 10.1073/pnas.1401884111 24889615 10.1073/pnas.1401884111 PMC4066499

[4] Doster MA, Sweigaird JA, Fry WE. The influence of host resistance and climate on the initial appearance of foliar late blight of potato from infected seed tubers. Am Potato J. 1989;66: 227–233.

[5] Fry WE. Phytophthora infestans: The plant (and R gene) destroyer. Molecular Plant Pathology. 2008 pp. 385–402. doi: 10.1111/j.1364-3703.2007.00465.x 18705878 10.1111/j.1364-3703.2007.00465.x PMC6640234

[6] Grenville-Briggs LJ, van West P. The Biotrophic Stages of Oomycete-Plant Interactions. In: Laskin AI, Bennett JW, Gadd GM, editors. Advances in Applied Microbiolgy. vol. 75. 2005. pp. 217–243. 10.1016/S0065-2164(05)57007-2 16002014

[7] Shakya SK, Goss EM, Dufault NS, van Bruggen a HC. Potential effects of diurnal temperature oscillations on potato late blight with special reference to climate change. Phytopathology. 2015;105: 230–238. doi: 10.1094/PHYTO-05-14-0132-R 25140388 10.1094/PHYTO-05-14-0132-R

[8] Haverkort AJ, Boonekamp PM, Hutten R, Jacobsen E, Lotz LAP, Kessel GJT, et al Societal costs of late blight in potato and prospects of durable resistance through cisgenic modification. Potato Research. 2008 pp. 47–57.

[9] Forbes GA, Fry WE, Andrade-Piedra JL, Shtienberg D, Ciancio A, Mukerji KG. Simulation Models for Potato Late Blight Management and Ecology. Integrated Management of Diseases Caused by Fungi, Phytoplasma and Bacteria. 2008 pp. 161–177.

[10] Wallin JR. Summary of recent prorgess in predicting late blight epidemics in United States and Canada. Am Potato J. 1962;39: 306–312.

[11] Fry WE, Apple AE, Bruhn JA. Evaluation of potato late blight forecasts modi- fied to incorporate host resistance and fungicide weathering. Phytopathology, 73, 1054–1059. Garrett. Phytopathology. 1983;73: 1054–1059.

[12] Grünwald NJ, Potato CEC, Blight L, México E De, Pathology P, Rubio-covarrubias OA, et al Potato Late-Blight Management in the Toluca Valley: Forecasts and Resistant Cultivars. Plant Dis. 2000;84: 410–416. 10.1094/PDIS.2000.84.4.410 30841162

[13] Grünwald NJ, Fry WE. Potato Late Blight Management in the Toluca Valley: Field Validation of SimCast Modified for Cultivars with High Field Resistance. Plant Dis. 2002; 1163–1168. 10.1094/PDIS.2002.86.10.1163 30818512

[14] Hijmans RJ, Forbes GA, Walker TS. Estimating the global severity of potato late blight with GIS-linked disease forecast models. Plant Pathol. 2000;49: 675–705.

[15] Sparks AH, Forbes G, Hijmans R, Garrett K. Climate change may have limited effect on global risk of potato late blight. Glob Chang Biol. 2014;20: 3621–31. doi: 10.1111/gcb.12587 24687916 10.1111/gcb.12587

[16] Krause RA, Massie LB, Hyre RA. Blitecast: a computerized forecast of potato late blight. Plant Dis Report. 1975;59: 95–98.

[17] Wiik L. Potato Late Blight and Tuber Yield: Results from 30 Years of Field Trials. Potato Res. 2014;57: 77–98.

[18] Hannukkala AO, Kaukoranta T, Lehtinen A, Rahkonen A. Late-blight epidemics on potato in Finland, 1933–2002; increased and earlier occurrence of epidemics associated with climate change and lack of rotation. Plant Pathol. 2007;56: 167–176.

[19] Jordbruksverket. Föreskrifter om ändring i Statens jordbruksverks föreskrifter (SJVFS 1995:90) om certifiering m.m. av utsädespotatis [Internet]. 2015. Available: http://www.jordbruksverket.se/download/18.b0ed33315186da117d2542/1449668007573/2015-039.pdf

[20] European and Mediterranean Plant Protection Organization. EPPO Standards Certification Schemes Seed Potatoes PM 4/28(1). 1999.

[21] EPPO. Efficacy evaluation of fungicides & bactericides. EPPO Standards PP1. 2004. pp. 6–8.

[22] Organization E and MPP. EPPO Standards. Efficancy Evaluation of Plant Protection Products. Fungicides and Bactericides, vol 2. 2004.

[23] Liljeroth E, Lankinen Å, Wiik L, Burra DD, Alexandersson E, Andreasson E. Potassium phosphite combined with reduced doses of fungicides provides efficient protection against potato late blight in large-scale field trials. Crop Prot. 2016;86: 42–55.

[24] Hurley P, Edwards M, Luhar A. Evaluation of TAPM V4 for Several Meteorological and Air Pollution Datasets. Air Qual Clim Chang. 2009;43: 19–24.

[25] Tang L, Miao J, Chen D. Performance of TAPM against MM5 at urban scale during GÖTE2001 campaign. Boreal Environ Res. 2009;14: 338–350.

[26] Kalnay E, Kanamitsu M, Kistler R, Collins W, Deaven D, Gandin L, et al The NCEP/NCAR 40-year reanalysis project. Bull Am Meteorol Soc. 1996;77: 437–470.

[27] Wang E, Engel T. Simulation of Phenological Development of Wheat Crops. Agric Syst. 1998;58: 1–24.

[28] Lee ET. Statistical Methods for survival data analysis. Belmond: Wadsworth Inc.; 1980.

[29] Bradburn MJ, Clark TG, Love SB, Altman DG. Survival Analysis Part II: Multivariate data analysis–an introduction to concepts and methods. Br J Cancer. 2003;89: 431–436. doi: 10.1038/sj.bjc.6601119 12888808 10.1038/sj.bjc.6601119 PMC2394368

[30] Pulatov B, Linderson M-L, Jönsson AM. Pulatov B., Hall K., Linderson M.-L. & Jönsson A. M. (2015). Modelling climate change impact on potato crop, the risk of frost damage and heat stress in Northern Europe. Agricultural and Forest Meteorology 214–215:281–292. Agric For Meteorol. 2015;214–215: 281–292.

[31] Bradburn MJ, Clark TG, Love SB, Altman DG. Survival Analysis Part III: Multivariate data analysis–choosing a model and assessing its adequacy and fit. Br J Cancer. 2003;89: 605–611. doi: 10.1038/sj.bjc.6601120 12915864 10.1038/sj.bjc.6601120 PMC2376927

[32] Niederhauser JS. Phytophthora infestans: tile Mexican connection In: Lucas JA, Shattock RC, Shaw DS, Cooke LR, editors. Phytophthora. Cambridge: Cambridge Univ. Press; 1991 pp. 25–45.

[33] Grünwald NJ, Flier WG. The Biology of Phytophthora infestans at its centre of origin. Annu Rev Phytopathol. 2005;43: 171–190. doi: 10.1146/annurev.phyto.43.040204.135906 16078881 10.1146/annurev.phyto.43.040204.135906

[34] Kadir S, Umaerus V. Phytophthora infestans A2 compatability type recorded in Sweden. Book of Abstracts, 10th Triennial Conference EAPR, In: Aalborg, Denmark. 1987. p. 223.

[35] Hermansen A, Hannukkala A, Hafskjoldf-Nærstad R, Brurberg MB. Variation in populations of Phytophthora infestans in Finland and Norway: Mating type, metalaxyl resistance and virulence phenotype. Plant Pathol. 2000;49: 11–22.

[36] Brurberg MB, Elameen A, Le VH, N??rstad R, Hermansen A, Lehtinen A, et al Genetic analysis of Phytophthora infestans populations in the Nordic European countries reveals high genetic variability. Fungal Biol. 2011;115: 335–342. doi: 10.1016/j.funbio.2011.01.003 21530915 10.1016/j.funbio.2011.01.003

[37] Brurberg MB, Hannukkala A, Hermansen A. Genetic variability of Phytophthora infestans in Norway and Finland as revealed by mating type and fingerprint probe RG57. Mycol Res. 1999;103: 1609–1615.

[38] Yuen JE, Andersson B. What is the evidence for sexual reproduction of Phytophthora infestans in Europe? Plant Pathology. 2013 pp. 485–491.

[39] Lehtinen A, Hannukkala A. Oospores of phytophthora infestans in soil provide an important new source of primary inoculum. Agric Food Sci. 2004;13: 399–410.

[40] Sjöholm L, Andersson B, Högberg N, Widmark A-K, Yuen J. Genotypic diversity and migration patterns of Phytophthora infestans in the Nordic countries. Fungal Biol. 2013;117: 722–30. doi: 10.1016/j.funbio.2013.08.002 24119411 10.1016/j.funbio.2013.08.002

[41] Haas BJ, Kamoun S, Zody MC, Jiang RHY, Handsaker RE, Cano LM, et al Genome sequence and analysis of the Irish potato famine pathogen Phytophthora infestans. Nature. 2009;461: 393–8. doi: 10.1038/nature08358 19741609 10.1038/nature08358

[42] Minogue KP, Fry WE. Effect of temperature, relative humidity, and rehydration rate on germination of dried sporangia of Phytophthora infestans. Phytopathology. 1981 pp. 1181–1184.

[43] Harrison JG, Lowe R. Effects of humidity and air speed on sporulation of Phytophthora infestans on potato leaves. Plant Pathol. 1989;38: 585–591.

[44] Mariette N, Mabon R, Corbiere R, Boulard F, Glais I, Marquer B, et al Phenotypic and genotypic changes in French populations of Phytophthora infestans: are invasive clones the most aggressive? Plant Pathol. 2016;65: 577–586.

[45] Clement JAJ, Magalon H, Glais I, Jacquot E, Andrivon D. To Be or Not to Be Solitary: Phytophthora infestans ‘ Dilemma for Optimizing its Reproductive Fitness in Multiple Infections. PLoS One. 2012;7: e37838 doi: 10.1371/journal.pone.0037838 22675493 10.1371/journal.pone.0037838 PMC3365895

[46] Scherm H, van Bruggen AHC. Sensitivity of simulated dew duration to meteorological variations in different climatic regions of California. Agric For Meteorol. 1993;66: 229–245.

[47] Scherm H, van Bruggen AHC. Global warming and nonlinear growth: how important are changes in average temperature? Phytopathology 84:1380–1384. Phytopathology. 1994;84: 1380–1384.

[48] Ewers BE, Oren R. Analyses of assumptions and errors in the calculation of stomatal conductance from sap flux measurements. Tree Physiol. 2000;20: 579–589. 12651422 10.1093/treephys/20.9.579

[49] Schwarz G. Estimating the Dimension of a Model. Ann Stat. 1978;6: 461–464.

[50] Chakraborty S. Migrate or evolve: Options for plant pathogens under climate change. Global Change Biology. 2013 pp. 1985–2000. doi: 10.1111/gcb.12205 23554235 10.1111/gcb.12205

