# Supplementary material for: Earlier occurrence and increased explanatory power of climate for the first incidence of potato late blight caused by Phytophthora infestans in Fennoscandia
Source: PLoS One. 2017 May 30;12(5):e0177580. doi: 10.1371/journal.pone.0177580 (PMC5448744; doi:10.1371/journal.pone.0177580)
Supplement: S1 Fig — (PDF) [file pone.0177580.s001.pdf]

Sensitivity of estimation of incidence of late blight.

The first incidence of late blight was estimated using the percentage of cover of late blight when first scored (a reduction of DAP by three days was made if the attack at the first assessment was < 0.01%, by five days if the attack was > 0.01-0.099%, by seven days if the attack was 0.1-1.0% and by nine days if the attack was > 1%; see methods). Scorings were made at least once a week, in most cases more often. Since no daily scorings are available this introduces an uncertainty which we investigate by adding a random component (± 5 days) into the estimated first date of occurrence.

The following figures show distribution of the p-values of all parameters listed in table one with a randomised first occurrence of (± 5 days) using 1000 randomised datasets.

In all randomised datasets, the model containing the product of the beta temperature with the relative humidity has the highest explanatory value superior. The last plot lists the p-value of the parameter minimum temperature of a model containing the product of the beta temperature with the relative humidity and the minimum temperature.

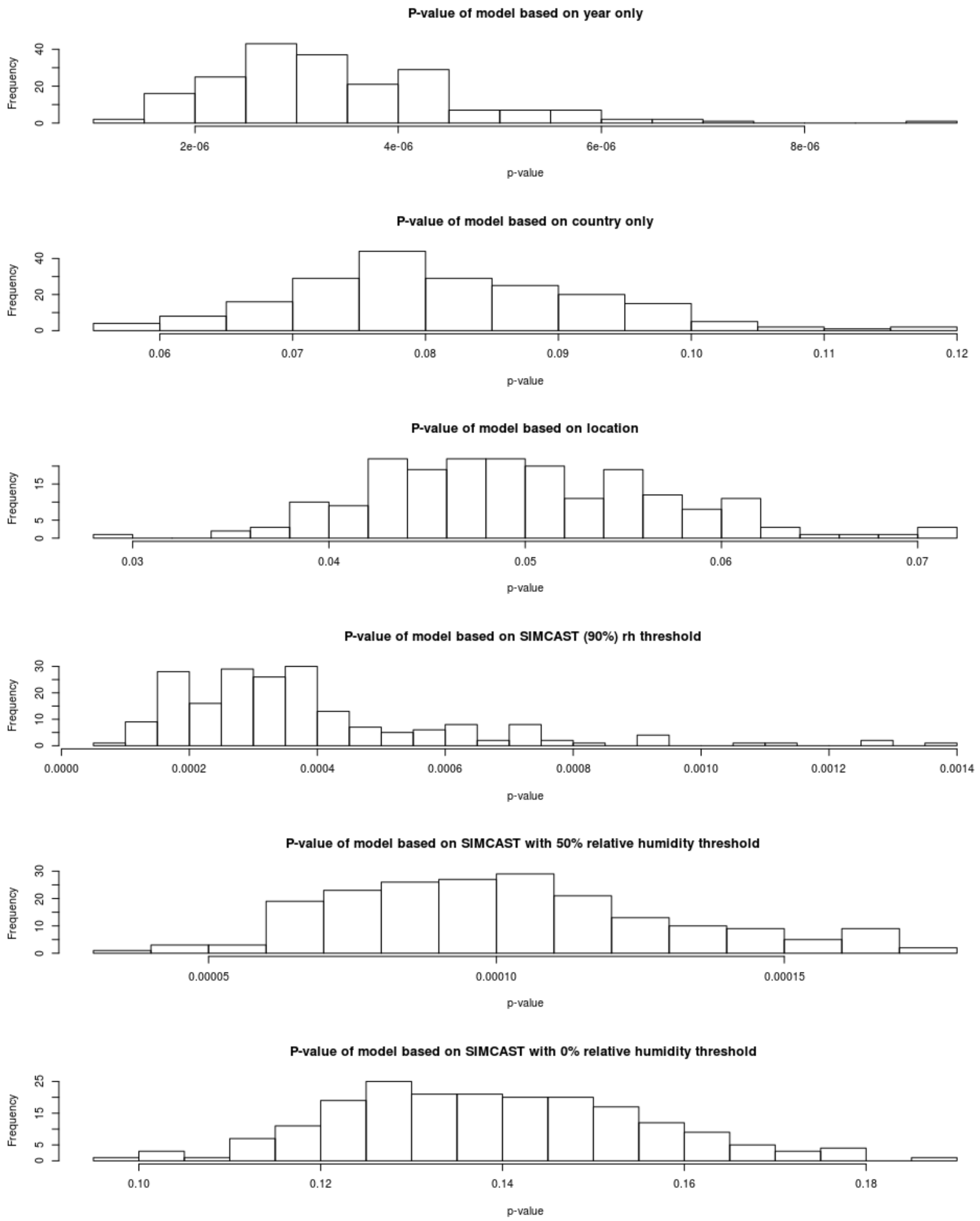
P-value of model based on year only
Frequency
p-value
P-value of model based on country only
Frequency
p-value
P-value of model based on location
Frequency
p-value
P-value of model based on SIMCAST (90%) rh threshold
Frequency
p-value
P-value of model based on SIMCAST with 50% relative humidity threshold
Frequency
p-value
P-value of model based on SIMCAST with 0% relative humidity threshold
Frequency
p-value

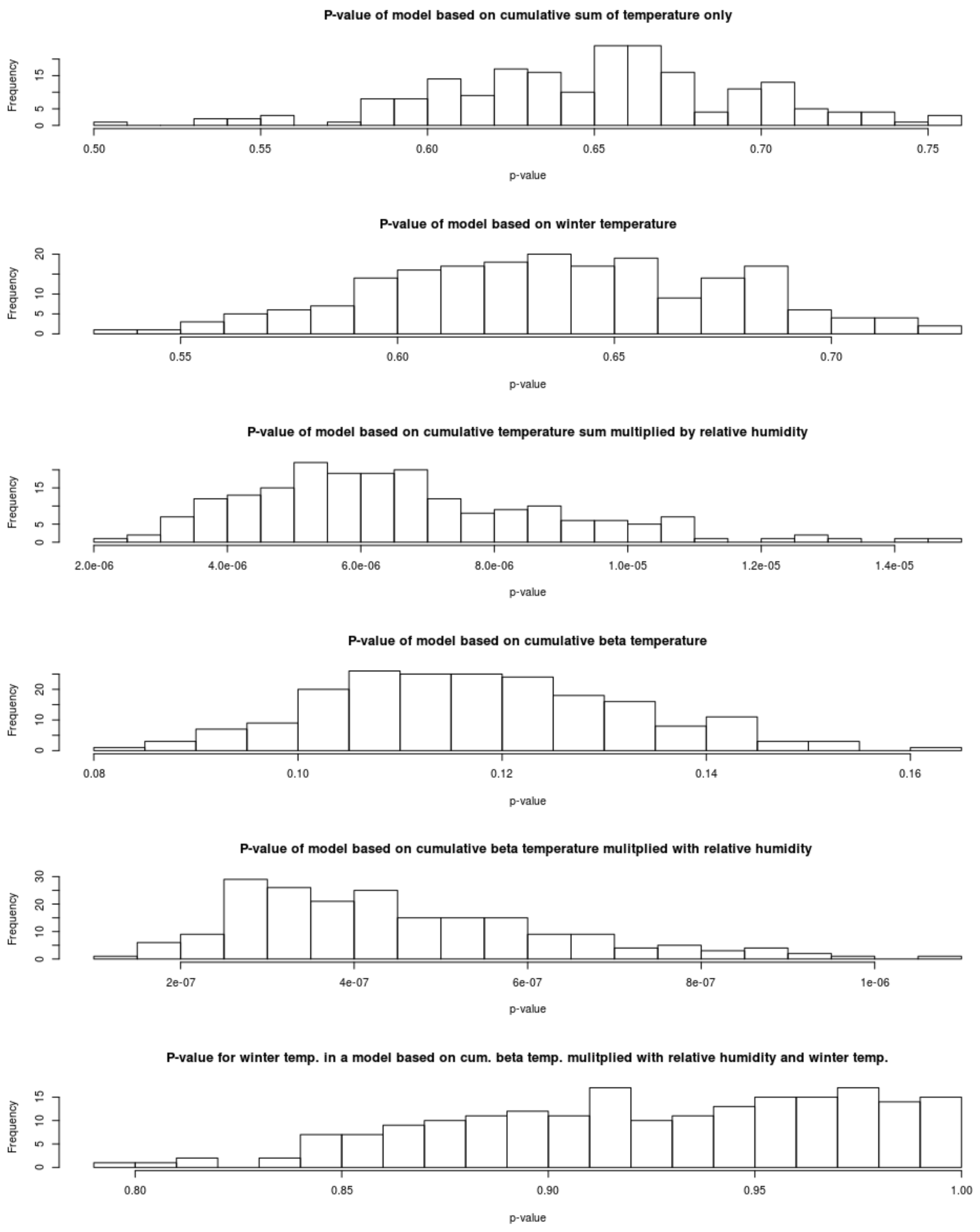

Fig. S1. Distributions of p-values resulting from of added uncertainty in day of incidence of late blight.
