# Supplementary material for: Earlier occurrence and increased explanatory power of climate for the first incidence of potato late blight caused by Phytophthora infestans in Fennoscandia
Source: PLoS One. 2017 May 30;12(5):e0177580. doi: 10.1371/journal.pone.0177580 (PMC5448744; doi:10.1371/journal.pone.0177580)
Supplement: S2 Fig — (PDF) [file pone.0177580.s002.pdf]

Uncertainty of simulated climate variables for the Swedish sites

The observation data is collected from different station ranging over different time spans: Mosslunda 4 years, Lilla Böslid:7 years and Borgeby 8 years. Observed data has been collected from the LANTMET network and retrieved from http://www.ffe.slu.se/lm/LMHome.cfm.

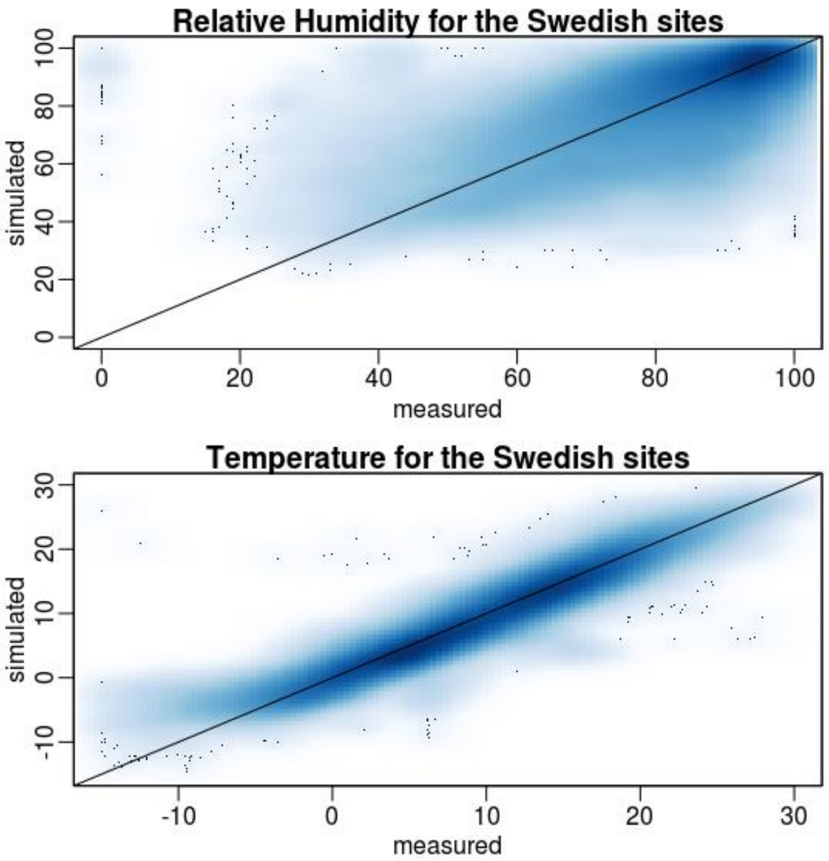

Fig. S2. Simulated versus measured relative humidity and temperature for the Swedish sites.
