# Supplementary material for: Earlier occurrence and increased explanatory power of climate for the first incidence of potato late blight caused by Phytophthora infestans in Fennoscandia
Source: PLoS One. 2017 May 30;12(5):e0177580. doi: 10.1371/journal.pone.0177580 (PMC5448744; doi:10.1371/journal.pone.0177580)
Supplement: S3 Fig — (PDF) [file pone.0177580.s003.pdf]

Description of the data

The table late_blight_observation_and_met_covariates.csv, available from the DataGURU server (dataguru.lu.se; with the DOI 10.18161/late_blight.201703) contains all data (observed late blight incidence and simulated weather) used in our analyses.

It is a table of daily values. The columns of the dataset are listed and described in table S1.

Table S1. Description of the daily data used for the analysis in the study “Earlier occurrence and increased explanatory power of climate for the first incidence of potato late blight caused by *Phytophthora infestans* in Fennoscandia” by Lehsten, Wiik, Hannukkala, Andreasson, Chen, Ou, Liljeroth, Lankinen and Grenville-Briggs. Currently under review in PlosONE.

Column number	Header	Description
1	days_since_planting	Days since planting
2	event	Whether late blight was detected on this day. (0/1)
3	year	Year of observation
4	station	The stations 1-3 are in Sweden, the remaining in Finland.
5	cumsimc	Cumulative SIMCAST blight units with the standard threshold of 90% relative humidity
6	csblight_50	Cumulative SIMCAST blight units with the standard threshold of 50% relative humidity

7	csblight_95	Cumulative SIMCAST blight units with the standard threshold of 95% relative humidity
8	csblight_0	Cumulative SIMCAST blight units with the standard threshold of 0% relative humidity
9	cs_mean_tas	Cumulative sum of mean daily temperature [°C]
10	cs_mean_rh	Cumulative sum of mean relative humidity [%]
11	cs_beta_tas	Cumulative sum of mean growth temperature [unit less], see methods for beta function
12	cs_beta_tas_t_mean_rh	Cumulative sum of mean growth temperature multiplied with the mean relative humidity
13	winter_min_temp	Minimum winter temperature of the winter prior the vegetation season.
